# Autism and Migraine: A Narrative Review

**DOI:** 10.7759/cureus.70060

**Published:** 2024-09-23

**Authors:** Ali Alsaad

**Affiliations:** 1 Clinical Neuroscience, King Faisal University, Al Ahsa, SAU

**Keywords:** autism, headache, migraine, pain, sensory processing

## Abstract

Both autism spectrum disorder and migraine are heterogeneous disorders from a genetic and phenotypic perspective. Both disorders impact the patient and caregiver’s quality of life. The link between autism spectrum disorder and migraine headaches has been suggested through some similarities in some genetic, structural, and psychopathological studies. However, few clinical studies looked into this association. The diagnosis of migraine in children and adolescents with autism is more challenging for several reasons, for example, the impairment in social communication that is a core feature in autism and has a high comorbidity with language disorders and/or intellectual delay. Pain expression or pain behavior is another challenging reason. This study aims to review the association between autism and migraine and to help improve the clinical assessment of children and adolescents with autism and comorbid migraine.

## Introduction and background

Autism spectrum disorder (ASD) is a heterogeneous neurodevelopmental disorder that is characterized by a persistent deficit in social communication and social interaction in the presence of restricted and repetitive behaviors, stereotyped movements, cognitive inflexibility, and unusual sensory sensitivity [1]. The prevalence of ASD in the general population is around 1%-1.5% [2,3].

Primary headache disorders are common in the pediatric population. They include migraines, tension-type headaches, and trigeminal autonomic cephalalgias [4]. Migraine is prevalent in the general pediatric population, and its prevalence increases throughout childhood, ranging from 1.4% to 3.2% in three- to seven-year-old children, 4%-11% in school-age children, and 8-28 % in teenage years [4-7]. Migraine does impact the quality of life via impacting the ability to participate in daily activities [4].

Migraine headache is more prevalent in children with ASD [8 ]. Migraine, however, is an underestimated diagnosis in children and adolescents with ASD. This review focuses on the shared pathophysiology between ASD and migraine. Additionally, this review aims to improve the medical care for children and adolescents with ASD by focusing on the clinical approach of migraine headaches in this population.

## Review

Genes/defective channel function

ASD and migraine are caused by complex multiple-genetic and non-genetic causes. Meaning both disorders are not caused by a single gene or genes.

Different studies have shown that one of the causes of ASD is related to channelopathy, specifically calcium voltage-gated channel subunit alpha1 (CACNA1 genes), sodium voltage-gated channel (e.g., SCN1A, SCN2A), and sodium/potassium pump gene (ATP1A2). Different subtypes were identified as a cause of autism and other psychiatric and medical disorders [9-11]. Channelopathy affects and alters crucial neural network processes in several brain areas causing several symptoms of ASD [10].

The missense mutations in three subtypes of these genes, CACNA1A, SCN1A, and ATP1A2, have also been found in patients with migraine, associated explicitly with family hemiplegic migraine [9].

It is important to keep in mind that ASD is a clinically complex neurodevelopmental disorder and impaired channelopathy is only one of several factors contributing to ASD [9]. The same thing applies to migraine.

Regarding genetics, a recent study done in 2019 [12] showed that having a first-degree relative with migraine headache was associated with an increase in the odds of having a child with ASD by 1.3-folds compared with those with unaffected first-degree relatives. Another study [13] found that the prevalence of migraine in children with ASD is increased compared to the general population. A retrospective study found in a retrospective study that migraine is the most common type of headache in adult patients with ASD referred to the neurology clinic [13,14].

Hyper-responsiveness (defensiveness)

The phenotype of autism and migraine also share some commonalities. Sensory processing disorder, specifically visual and/or auditory processing could be affected in both disorders.

Sensory processing studies have shown that different mechanisms and brain areas are involved in patients with photophobia/phonophobia.

Sensory discrimination helps us identify, distinguish, and interpret qualities of sensory input, and then the level or intensity of the response is regulated by sensory modulation. Sensorimotor integration is responsible for transforming sensations into motor responses. Sensory disorders, thus, could be classified into three types [15], sensory modulation disorders, sensory discrimination disorders, and sensorimotor integration disorders.

Sensory modulation disorder includes three sensory patterns [16]: hyporesponsiveness (under-reactive response to sensory stimuli), hyper-responsiveness (over-reactive, sensory under-responsiveness), and sensory seeking (craving with certain stimuli).

In this review, the focus would be on sensory hyper-responsiveness (sensory under-responsiveness) as an over-reactive response to a sensory stimulus is a common feature in both ASD and migraine.

Sensory hyper-responsiveness means that the response to a sensation would be longer in terms of duration, faster or more intense than those with typical sensory responsivity [17,18]. Hyperresponsivity may involve one sensory system or multiple sensory systems. People with sensory hyper-responsiveness have difficulties making effective responses, especially in new situations or during transition. Their responses are unconscious and automatic physiologic reactions to the sensation. The behavioral response ranges from avoidance of stimuli, withdrawal passively to a more active, aggressive, or impulsive response [15]. Sensory hyper-responsiveness can occur in combination with other patterns of sensory modulation disorders or with different types of sensory disorders.

From a structural and functional perspective, the thalamus plays a crucial role in multimodal sensory-motor processing. It relays and modulates information between cerebral cortical regions. In both, ASD and migraine, there is evidence of increased connectivity of the thalamus and alteration of function of auditory, visual, and olfactory cortices [19-22]. This could explain part of the etiology of photophobia/phonophobia.

Migraine in children with ASD, anxiety, and sensory hyper-responsiveness

The risk of anxiety disorder is increased in individuals who are diagnosed with ASD [23-25]. The relationship between sensory hyper-responsiveness and anxiety has been shown in different studies [26-28]. Various theories have been proposed regarding the direction of this relationship speculating whether anxiety in individuals with ASD would cause sensory hyper-responsiveness [29] or vice-versa [30].

A study [31] found that children with autism and sensory under-responsiveness with comorbid anxiety are more likely to experience migraine headaches. In this study, the authors suggested that this could be related to a subtype of ASD. In this study, the power was small, and specific symptoms of disorders (anxiety, migraine headache, and ASD) were not presented.

Sleep, ASD, and migraine

Sleep is commonly affected in individuals with ASD where around 66%-86% have reported having sleep disturbances [32,33]. Sleep disturbances affect the behavior and cognitive functioning of individuals with ASD.

Sleep disturbances are also known as a provoking factor for migraine attacks in individuals who are diagnosed with migraine.

Serotonin, ASD, and migraine

Dysregulation of the brainstem serotonergic system has been reported in both disorders [34,35]. Serotonin plays a vital role during brain development, and its dysregulation could lead to changes in thalamocortical connectivity. As mentioned before, also both disorders have been found to have increased thalamocortical connectivity [16,19,21,24,31].

Excitatory-inhibitory balance

It has been found in different studies that one of the pathophysiologies of ASD, as well as migraine, is an imbalance between cortical excitation and inhibition leading to hyperexcitability of cortical circuits [36-38].

Clinical assessment of migraine in children and adolescents with ASD

The assessment of migraine headaches is challenging in typically developed children, especially those who are younger than seven years [39].

Diagnosing migraine in children with ASD would be more challenging as they might have difficulty communicating the symptoms or are unable to do so, especially when ASD symptoms are more severe or when it is comorbid with another neurodevelopmental disorder. Migraine presents in different forms, such as migraine with/without aura, abdominal migraine, cyclic vomiting syndrome, FHM, and others. Each type has different symptoms and criteria for diagnosis. However, most of the symptoms in these subtypes are not specific. Another challenge is that symptoms might present differently in children and adolescents with ASD. For example, pain might present as self-injurious behavior (SIB), disruptive behavior, or deterioration in the overall adaptive level. It is difficult to apply migraine diagnostic criteria (International Classification of Headache Disorders 3rd Edition - ICHD-3) on children younger than seven years old [39] and more difficult to apply for children and adolescents with ASD.

The approach would be similar to approaching pain of unknown origin or approaching SIB in children and adolescents with ASD. All medical causes need to be rolled out, so the involvement of different specialists is required, such as a developmental pediatrician, dentist, ophthalmologist, otolaryngologist, neurology, and maybe gastrointestinal. The challenge comes after the results of all medical assessments and investigations are negative. Then, the source of the behavior most of the time is considered functional, and the treament sthifts to applied behavioral analysis (ABA). Even though it is challenging to diagnose a headache, it still should be included in the differential diagnosis. The diagnosis of migraine is deferential to other medical disorders, as well as to functional behavioral issues as there is no subjective test to diagnose it.

ABA could be of help to the neurologist to diagnose migraine, so here I recommend that they continue working as a team even after rolling out other medical or neurological causes of the current presentation of pain, SIB, disruptive behavior, or deterioration in the overall adaptive level.

History taking

Migraine headache occurs as a result of different bio-psycho-social factors, so migraine in children with ASD should be approached as such.

Taking a thorough history from parents/caregivers is, and should include, the child’s medical and mental health history, detailed sleep history, medications, family history of migraine, and triggers.

Migraine could be triggered and exacerbated by different factors. This includes specific food, environmental conditions such as weather changes, social stressors, and certain sensory stimuli such as strong smell or light. Lack of sleep and/or hormonal changes also could trigger a migraine headache. When the child undergoes an ABA assessment, looking at triggers not only from a functional behavior assessment but also for a possibility of migraine. This could help in directing the management and prevention of further migraine attacks and whatever negative impact they cause on the behavior of the child with ASD.

Another important trigger for migraine is anxiety [40-42]. As it is mentioned before, anxiety is highly comorbid with ASD, and it is also highly comorbid with migraine. Therefore, it is crucial to assess the child with ASD for comorbid mental illnesses.

Pain assessment tools

Pain-Assessment Tools are made for the assessment of behavioral pain in non-verbal children with neurological impairments and SIB. These tools might be of help in assessing pain caused by migraine in children and adolescents with ASD. As they require detailed information from parents, caregivers, and home-based nurses. An example of a pain-assessment tool is revised Face, Legs, Activity, Cry, Consolability scale (r-FLACC) and the Individualized Numeric Rating Scale (INRS). These 2 rating scales have the advantage of individualization, i.e., parents/ caregivers can indicate behaviors that are specific to the child. [43]

Physical examination

Physical examination is essential to roll out medical problems and to look for red flags that are suggestive of secondary headache causes. Common differential medical causes include dental problems, otitis media, sinusitis, the presence of a foreign body, visual issues, and papilledema [44,45]. A complete neurological examination is essential also to look for secondary causes of headache.

Diagnostic testing

There is no blood or imaging test to diagnose migraine. Diagnostic tests should be requested when required to investigate other causes of headaches. Imaging is necessary when signs of space-occupying lesions “red flags” are present. For examples, these red flags are the duration of suspected headache that is less than six months, abnormal findings in the neurological exam, vomiting, lack of family history of migraine, confusion, and sleep-related headaches [46].

The American Academy of Neurology (AAN) practice parameter advises against routine lab studies, lumbar puncture, EEG, and neuroimaging in patients without red flags and a normal neurologic exam [47]. However, in children with ASD who lack the ability to communicate, these practice parameters might or might not be applicable.

Treatment

Treatment for migraine in children with ASD would be patient-focused. ABA would be of help in identifying triggers and in modifying or changing negative behaviors resulting from the headache or the reinforcement wither positive or negative. Medications are usually indicated in the acute phase of migraine headache and might help in preventing further migraine attacks.

## Conclusions

Primary headache, especially migraine, is one of the most common neurological disorders in the pediatric population. The prevalence of migraine in children with ASD is increased compared to the general population. However, there is a lack of research regarding migraine in children and adolescents with ASD, in terms of assessment, diagnosis, and management.

Migraine should be considered in the differential diagnosis whenever a child or an adolescent with ASD presents with self-injurious behavior, disruptive behavior, or deterioration in the overall adaptive level. As migraine is a diagnosis of exclusion, other medical causes need to be rolled out. Applied behavior analysis could be of asset to the neurologist in diagnosing migraine in children with ASD.
